# An atypical abdominal manifestation of retroperitoneal teratoma: Case report

**DOI:** 10.1016/j.ijscr.2024.110605

**Published:** 2024-11-15

**Authors:** Umar Mahmood, Nabila Talat, Muhammad Usama Aziz, Muhammad Bilal Mirza, Warda Tahir, Samra Asif

**Affiliations:** a Department of Pediatric Surgery I, The University of Child Health Sciences and The Children's Hospital, Lahore, Pakistan; b Department of Pediatric Surgery, Gujranwala Medical College, Gujranwala, Pakistan

**Keywords:** Acute abdomen, Peritonitis, Teratoma

## Abstract

**Introduction & importance:**

Teratomas, typically situated in midline areas like the sacrococcygeal region, may rarely manifest in the retroperitoneal region. Often asymptomatic and incidentally discovered, they can lead to complications such as infection, tumor rupture, or, exceptionally, peritonitis.

**Case presentation:**

In a 2-year-old child with a history of trauma, an atypical presentation of acute abdomen and peritonitis was observed. Initial exploration of suspected visceral injury revealed a ruptured teratoma, successfully excised. Post-operatively, the patient remained stable and was discharged.

**Clinical discussion:**

Clinical presentation of RPTs ranges from simple abdominal pain to abdominal distension or a palpable abdominal mass. Some rare presentations include intraperitoneal hemorrhage or abscess formation. It can be quite challenging for surgeons and is notorious for iatrogenic injuries to surrounding structures. Patients with complete resection of a benign teratoma have an excellent prognosis.

**Conclusion:**

An asymptomatic, undiagnosed teratoma can cause an acute abdomen, as seen in our case where trauma led to tumor rupture, resulting in peritonitis and signs of abdominal distress necessitating prompt surgical intervention to avoid complications.

## Highlights

• Teratomas, typically situated in midline areas may rarely manifest in the retroperitoneal region.
• Often asymptomatic and incidentally discovered, they can lead to complications like infection, tumor rupture, or peritonitis.
• An undiagnosed asymptomatic teratoma may cause acute abdomen, tumor rupture, peritonitis, and abdominal distress.

## 1 Introduction

Teratomas and other germ cell tumors (GCTs) are neoplasms that are relatively prevalent in children. They can manifest in both gonadal and extragonadal sites, with the specific locations and varying tumor types based on the child's age. From a histological perspective, these tumors are characterized by the presence of tissues originating from the three germ cell layers: ectoderm, mesoderm, and endoderm. These tissues are foreign to the location in which they are found. In terms of morphology, teratomas may exhibit a solid, cystic, or mixed structure. While these tumors can develop in various parts or organs of the body, they are typically found in sacrococcygeal (40 %), gonadal (ovarian and testicular 27 %), mediastinal/cervicofacial (18 %) and intracranial (5 %) locations [1]. Retroperitoneal teratomas are rare and constitute only 3–4 % of all teratomas [2].

## 2 Methods

“This case report has been reported in line with the Surgical Case Report (SCARE) 2023 criteria.” [3].

## 3 Case history

**Fig. 1:**
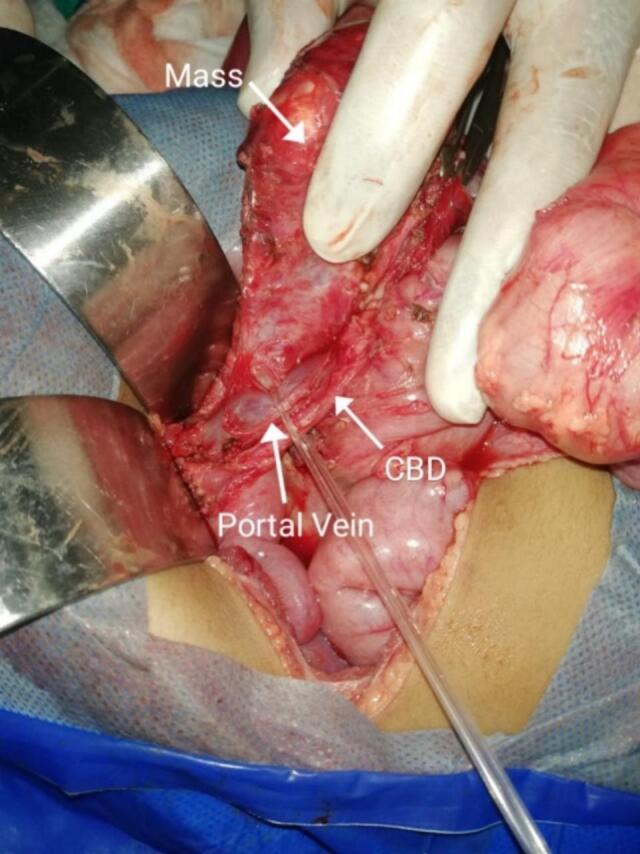
Subhepatic mass bounded laterally by common bile duct, portal vein, inferiorly by second part of duodenum, medially by inferior vena cava and superiorly by porta hepatis.

**Fig. 2:**
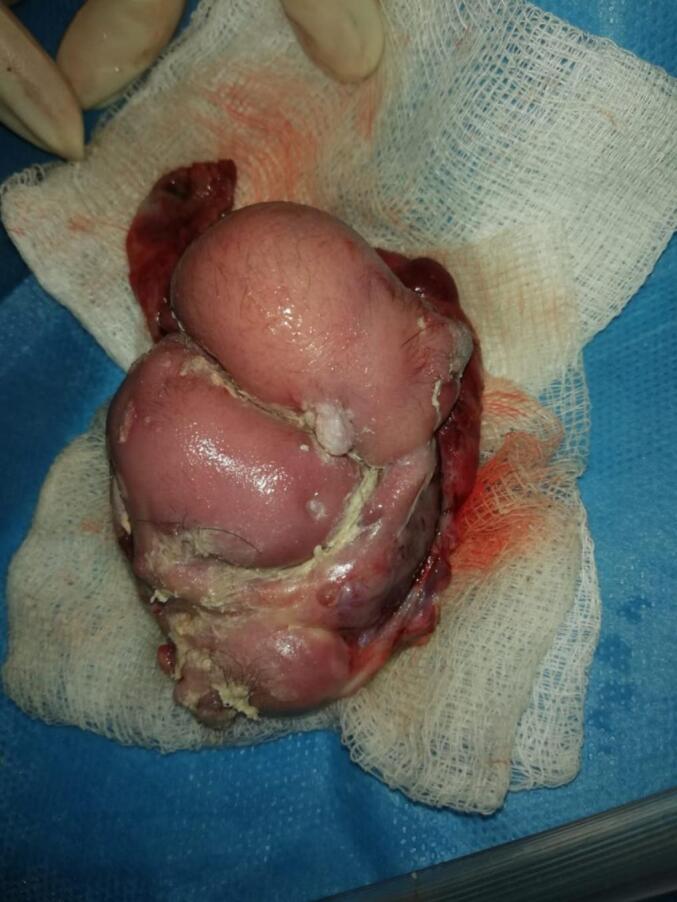
Excised mass giving an impression of feto-In fetus type teratoma but came out as mature teratoma.

**Fig. 3:**
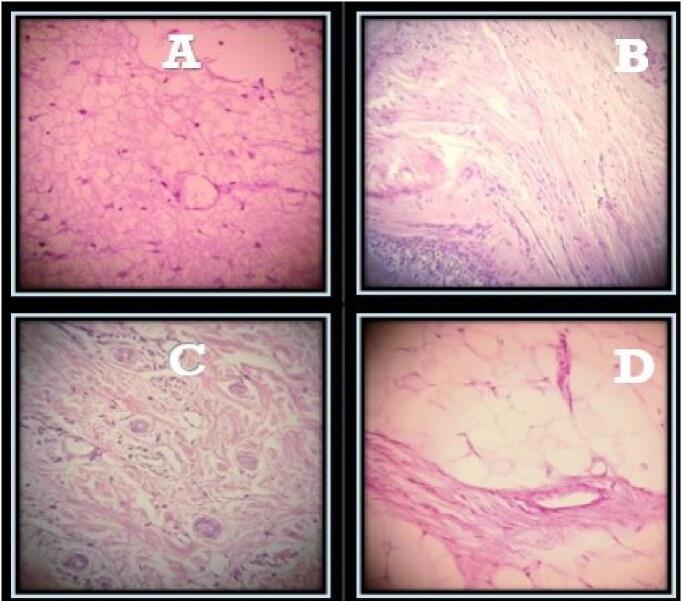
Pathologic examination of the mass with H/E staining demonstrating glial cells (A), mucosal cyst lining (B), dermal appendages (C) and adipocytes (D).

A Two-year-old male child presented to pediatric surgical emergency having complaints of abdominal distension, bilious vomiting and constipation from last two days. He had two days back history of falling from stairs over abdomen. The patient was managed initially for two days at the local hospital and then referred to our hospital on grounds of acute abdomen. On presentation in A &E department, his vitals were as follows: Pulse 118/min, R.R. 38/min, B.P. 90/60 mmHg and Temp was 98′F. On the General Physical Examination, the patient was dehydrated, irritable with GCS 15/15 and there was no jaundice, clubbing or pallor. On abdominal examination, it was distended with shiny skin (no imprint mark), tense and tender in all quadrants with absent bowel sounds. On digital rectal examination, rectum was empty. Based on history and examination, provisional diagnosis of peritonitis due to hollow viscera perforation was made. Patient was optimized and resuscitated as per ATLS protocols. After stabilization, X-ray Abdomen erect/supine and USG Abdomen were done. X-ray Abdomen depicted multiple air fluid levels, but no air under diaphragm. The USG abdomen revealed free fluid in abdomen with no solid visceral injury. CT scan was not done at that time due to patient critical condition. After optimization, surgery was planned with the provisional diagnosis of hollow visceral injury. Under aseptic measures, the right sided supraumbilical transverse incision was made and the abdomen was opened. The following per operative findings were noted: 600 ml (about 20.29 oz) of thick cheesy material mixed with purulent fluid, containing multiple small hairy particles in peritoneal cavity. A cystic structure was found in sub hepatic space at the level of porta hepatis which was a perforated sac having a mixed density mass of about 8x8x10 cm (about 3.94 in), bounded laterally by common bile duct, portal vein, inferiorly by second part of duodenum, medially by inferior vena cava and superiorly by porta hepatis (Fig. 1). It was retroperitoneal in origin. Meticulous dissection was done, and mass was excised completely. On gross examination of the mass, it seemed like a fetus-in-fetu type of teratoma with appearance of limb buds (Fig. 2). Excised mass was sent for histopathology to confirm the diagnosis. Post operatively patient was shifted to ICU. Post-operative recovery was smooth; oral started after 24 h as patient had purulent peritonitis and was discharged on 5th post operative day. Culture sensitivity of purulent fluid showed no growth, cytology showed absence of malignant cells, and a histopathology report revealed it as mature teratoma (Fig. 3). The patient had one follow-up after the surgery and then the follow-up was lost as the patient changed their contact number and never again followed up outdoors. On that visit, the patient had no active issue; the wound was healthy.

## 4 Discussion

Retroperitoneal teratoma (RPT) is an uncommon congenital tumor. Its occurrence ranges from 0.3 % to 3 % among all tumors and from 1 % to 10 % among primary retroperitoneal tumors in children [4]. The primary manifestation included an enlarged abdomen with the notable presence of a palpable mass [5]. Most common location of RPTs is suprarenal on left side with female predominance (Male to female ratio of 1: 2.7) but it may go otherwise; laterality may depend on age [6]. Although RPTs constitute a rare form of teratomas, especially in the pediatric population, no case was reported where trauma led to rupture, to the best of our knowledge, but a ruptured teratoma following infection in 8-month-old girl was reported that ultimately ended up in emergency laparotomy like our case report [7].

Clinical presentation of RPTs ranges from simple abdominal pain to abdominal distension or a palpable abdominal mass. Some rare presentations include intraperitoneal hemorrhage or abscess formation. Majjiga et al. reported a neonate with RPT who presented with hemorrhagic shock and massive abdominal distension 15 min after delivery [8]. Another case of A 9-year-old Polynesian girl presented with an abdominal mass and peritonitis, leading to exploratory laparotomy revealing a subhepatic abscess drained surgically, who then again presented with abdominal fullness, fever and chills after fifteen years due to recurrent infected RPT [9]. The presentation of RPT in our case was unique. Patient had a history of blunt abdominal trauma due to fall from stairs which led to rupture of his RPT, which subsequently released the cystic component of RPT into the peritoneum, causing signs and symptoms of peritonitis.

In diagnosis of RPT, radiological aid like ultrasonography and CT scan can help in establishing the diagnosis but definite diagnosis is made after surgical resection and careful histological evaluation [10]. Our patient was presented in A&E after trauma with provisional diagnosis of hollow visceral injury, so CT scan was not done at that time due to patient critical condition and ultrasonologist was also unable to comment on any abdominal mass. Our case was a post-traumatic rupture of RPT, a unique and unreported case, it was a challenge for us to decide pre-operatively whether to go for CT scan following resuscitation prior to Surgery.

Complete surgical excision is the mainstay of treatment for diagnosed cases of RPT [11]; in our case, it was a post-traumatic rupture of retroperitoneal teratoma that was not diagnosed prior to surgery as a patient was asymptomatic and it was incidental finding following blunt trauma to abdomen. So, surgical exploration and excision was done in these circumstances as the patient had established peritonitis.

Retroperitoneal teratoma excision is quite challenging for surgeons and is notorious for iatrogenic injuries to surrounding structures. Iatrogenic injuries to surrounding structures have been reported in about 50 % of cases [11]. Majjiga's neonate had a cauliflower-shaped tumor with hemorrhagic fluid was visible occupying most of the abdominal cavity but clearly originating from the retroperitoneum which was completely excised [8]. A Polynesian girl with recurrent infected RPT had a 5300-g mass adherent to the inferior vena cava, excised completely [9]. In our case a very rare presentation in sub hepatic space at level of porta hepatis has been observed. It was bounded by various vital structures such as Common Bile duct, hepatic artery, portal vein and Inferior Vena cava. Meticulous dissection was done to excise the whole mass without injury to surrounding structures.

Histopathology in most cases of RPT is mature teratoma. Only 1 % of all teratomas have immaturity or malignancy [12]. Neonate with hemoperitoneum had lobulated mass excised which appeared to be an immature teratoma on histopathology [8]. Pathological examination of recurrent infected RPT confirmed a mature, cystic teratoma containing hair, caseous material, and fluid with bacterial growth [9]. Histopathology in our case also received as mature teratoma.

Patients with complete resection of a benign teratoma have an excellent prognosis. Chemotherapy does have a role in the management of malignant teratomas, however, when possible, surgical resection with negative margins should be performed [13]. Mature teratomas have a good prognosis and complete surgical excision is enough [14].

## 5 Conclusions

An asymptomatic teratoma may be present as an acute abdomen following trauma. Prompt surgical intervention is essential to prevent further complications. Surgeons must ensure thorough exploration and complete tumor excision while carefully avoiding injuries to surrounding vital structures.

## Consent

Informed consent was taken from the parents.

## Ethical approval

Ethical approval for this study was provided by the Institutional Review Board of The University of Child Health Sciences, and The Children's Hospital, Lahore, Pakistan on 24 March2023.

## Guarantor

Dr. Umar Mahmood.

## Research registration number

1. Name of the registry: N/A.
2. Unique identifying number or registration ID: N/A.
3. Hyperlink to your specific registration (must be publicly accessible and will be checked): N/A.

## Funding

The authors received no financial support for the research, authorship, and publication of this article.

## Author contribution

Study concept or design: NT, UM, MUA, MBM.

Data collection: UM, WT, SA.

Data analysis or interpretation: UM, MUA, WT.

Writing the paper: NT, UM, MUA, MBM.

## Conflict of interest statement

There is no conflict of interest.
